# The impact of goods and services tax increase on economic crime: Evidence from China’s tobacco tax hike

**DOI:** 10.18332/tid/197330

**Published:** 2025-01-17

**Authors:** Xuanxuan Zhang, Zili Zhang

**Affiliations:** 1 School of Public Finance and Taxation, Capital University of Economics and Business, Beijing, China; 2 School of Public Finance and Taxation, Zhejiang University of Finance & Economics, Hangzhou, China; 3 Key Research Center of Philosophy and Social Sciences of Zhejiang Province, The Institute of Local Finance Research at Zhejiang University of Finance and Economics, Hangzhou, China

**Keywords:** GST increase, tobacco exercise tax, economic crime

## Abstract

**INTRODUCTION:**

Despite the acknowledged interconnection between socioeconomic environment and economic crime, research on the relationship between Goods and Services Tax (GST) and economic crime is scarce because of their complicated relationship. This study examines the impact of the GST increase on the illicit tobacco trade.

**METHODS:**

Based on China’s tobacco excise tax shock in 2015, this study employs a difference-in-difference (DID) method to analyze the impact of the GST increase on economic crime. Panel data used in this research are collected by combining prefecture-level socioeconomic data with smuggling data from 2011 to 2016, including variables such as economic crime, wages, GDP per capita, and population. Economic crime, our core dependent variable, is evaluated by the seized-illegal value of smuggling tobacco (SIVST). In order to estimate the heterogeneity effect of tax hikes by region, we classify prefectures into three groups and add a triple interaction term into the regression model.

**RESULTS:**

We find that economic crime will be further elevated in places where it was initially higher, stimulated by the excise tax hike. Specifically, areas with high per capita tobacco smuggling in 2014 showed a significant increase in cigarette crime following the 2015 cigarette excise tax increase. For every 1-unit increase in per capita illicit trade in cigarettes in the region in 2014, the local illicit trade increased by 0.25 units after 2015. Heterogeneity analysis shows that the impact of GST increase on the SIVST among prefectures is more pronounced among the coastal prefectures. At the same time, there is no significant difference between border and central prefectures.

**CONCLUSIONS:**

To mitigate the adverse effects of GST increases on economic crime, governments should implement measures to combat tobacco smuggling, particularly in regions with prevalent criminal activity. This is especially crucial when policymakers opt to raise tobacco taxes for fiscal purposes or tobacco control initiatives.

## INTRODUCTION

Economic crime, encompassing a range of illicit activities such as fraud, corruption, money laundering, and smuggling, has garnered increasing attention from economists, policymakers, and law enforcement agencies worldwide^1,2^. In recent years, the prevalence and complexity of economic crimes have posed significant challenges to economic development, financial stability, and social cohesion across nations. It not only undermines the integrity and efficiency of markets, distorting resource allocation and hindering economic growth, but also erodes investor confidence, impedes entrepreneurship, and deters foreign investment, thereby stifling innovation and productivity enhancement efforts^3^. Moreover, economic crime imposes substantial costs on society, including financial losses and socioeconomic consequences^4^. Victims of fraud, smuggling, and financial scams suffer direct monetary losses, while the broader population bears the indirect costs through increased taxes, reduced public services, and diminished trust in institutions. Given economic crime’s multifaceted nature and pervasive impact, understanding its influencing factors is essential for designing effective policy responses and strengthening regulatory frameworks.

Through empirical analysis and theoretical modeling, researchers paid much attention to the factors influencing economic crime. First, economic adversity, such as unemployment and poverty, is a significant factor contributing to higher rates of economic crime^5-7^. Second, economic crime is also affected by the legal and market environment. Market credit issues, power abuse, and disruption of economic order are directly connected to economic crimes^8^. Third, technological development is another factor. The evolution of technologies plays a crucial role in stimulating and preventing economic crimes. Technological advancements can enable more sophisticated methods of committing economic crimes and offer new tools for law enforcement and businesses to detect and prevent these activities. In addition, cultural and education policies are widely considered significant elements shaping criminal behavior^9,10^.

Although many researchers have analyzed the factors that influence economic crime, the impact of the Goods and Services Tax (GST) on it is always ignored. So far, researchers have not fully understood the relationship between GST and economic crime. On the one hand, criminal activity is rational behavior under uncertainty, depending on the expected benefits and costs of engaging in a criminal activity. Offenders decide to commit crimes if their expected benefits are greater than the expected costs of punishment. As the tax on a commodity increases, the price of it rises. Holding the punishment and economic conditions unchanged, a higher commodity price could make more profits for economic offenders from smuggling it.

On the other hand, an increase in the GST may result in increased government revenues, which means that the government will have more money to combat illicit trade. The increased price reduces the demand for tobacco, which may squeeze the profit from smuggling. Hence, the impact of the tax on economic crime is unclear. To disentangle the ambiguity, this study attempts to investigate how changes in tax affect changes in economic crime by employing a difference-in-difference (DID) method.

In China, the situation becomes particularly intriguing when focusing on economic crime and the growth of GST. Because of the severe wealth disparity and a large number of unemployed groups and low-income groups, China witnessed a high incidence of economic crime in the past decade^11-14^. For example, from 2023 to March 2024, Chinese police investigated and solved approximately 95000 cases related to economic offenses. Regarding GST growth, China has high GST rates, especially on goods like tobacco, intended to generate significant government revenue and curb the consumption of harmful products. However, they also create strong incentives for smuggling and other forms of economic crime, as Chinese individuals and organizations seek to avoid these taxes and profit from illegal trade.

This study focuses on the tobacco sector of China for the following reasons. First, the consumption tax on tobacco products in China is one of the highest among all commodities, reflecting the government’s efforts to curb tobacco use and generate substantial revenue. Second, the tobacco industry plays a major role in the Chinese economy. As the world’s largest producer and consumer of tobacco, China provides research findings that hold immense social value. Third, cigarette-related economic crime represents a substantial portion of overall economic crime in China. The high demand for tobacco products, coupled with substantial taxes, fuels the illicit trade in tobacco, including smuggling, which not only results in significant financial losses for the government but also poses a threat to public health and safety. This creates a unique context for studying the relationship between Chinese tax policies and economic crime.

Given the high stakes involved, including significant government revenue and public health considerations, deeply understanding the dynamics of tobacco-related economic crime in response to tax changes is crucial. After verifying the relationship between GST increase and economic crime, this article further examines the regional heterogeneity of the effect by investigating whether the effect is greater in the border and coastal cities compared with that in central cities because of their different convenience of commodity smuggling among the three groups of cities.

Therefore, this study could provide insights into the broader implications of GST changes on economic crime by examining how cigarette-related tax affects cigarette smuggling behavior. This informs the design of more practical regulatory frameworks and enforcement strategies, ultimately contributing to China’s more stable and transparent economic environment. On the one hand, the heterogeneity analysis suggests that as policymakers want to get more fiscal revenue by raising tax rates, the heterogeneously negative effect of tax hikes on economic crime should be considered. Setting distinct tax rates and adjusting the degree of economic crime crackdowns in different regions can effectively balance revenue generation with crime prevention. On the other hand, by shedding light on the mechanisms through which GST increases the impact of economic crime, this study can inform broader strategies for combating illicit trade and enhancing the overall integrity of the market. In addition, the findings from this research could have far-reaching implications. The interplay between tax policies and criminal behavior in China, which is found in this study, offers potential lessons for other countries facing similar challenges.

## METHODS

### Background

Before May 2015, the specific excise tax on cigarettes at the producer price level was 0.06 RMB per pack (1000 Chinese Yuan Renminbi about US$140, current exchange), and the *ad valorem* tax was 56% for cigarettes that cost (producer price) ≥7 RMB and 36% for cigarettes costing (producer price) <7 RMB per pack. At that period, a 5% *ad valorem* tax per pack was levied at the wholesale price level^15^. To curb the habit of smoking, in 2015, the State Administration of Taxation issued the Circular of the Ministry of Finance and State Administration of Taxation on Adjusting the Cigarette Consumption Tax [Caishui (2015) No. 60, ‘Circular 60’], announcing an increase in the *ad valorem* tax rate on cigarettes excise tax at the wholesale level from 5% to 11%. The policy was effective from 10 May 2015.

### Data and approach

This study employs a difference-in-difference (DID) method to analyze the impact of GST increase on economic crime. To achieve the research goals, we combine two primary data sources at the prefecture-year level during 2011–2016 to generate a unique dataset used to analyze the causal relationship between tax and economic crime. The first dataset provides annual information on SIVST at the prefecture level, which is manually collected from the China Tobacco Yearbook compiled by the State Tobacco Monopoly Administration (STMA)^16^. The STMA, to prevent the data from informing the tobacco control community, has stopped disclosing the data in the China Tobacco Yearbook since 2017. This is the primary reason for utilizing research data up to 2016. The second database is obtained from the China City Statistical Yearbook. Other required prefecture data, including some socioeconomic features of prefectures, are from this database.

### Variables

**Figure 1:**
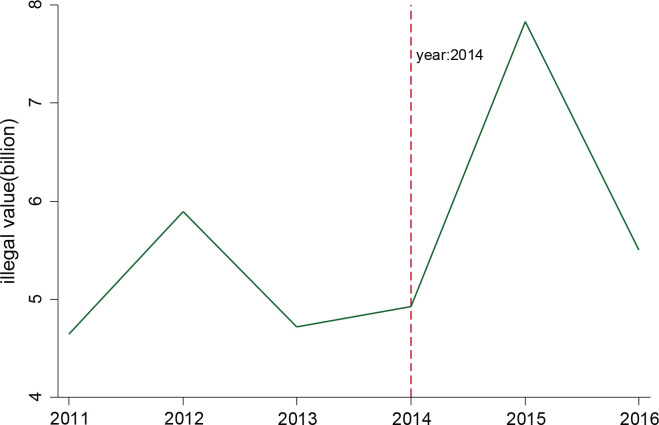
Aggregate annual seized-illegal value of smuggling tobacco for 2011–2016

Cigarette economic crime is this study’s primary outcome variable, measured by tobacco smuggling value. Figure 1 shows the trends in the seized-illegal value of smuggling tobacco (SIVST) for China from 2011 to 2016. SIVST gradually increased over time and, surprisingly, experienced a large spike in 2015, coinciding with a concurrent rise in the excise tax on tobacco. National SIVST in 2015 was almost two times that in 2014.

**Figure 2:**
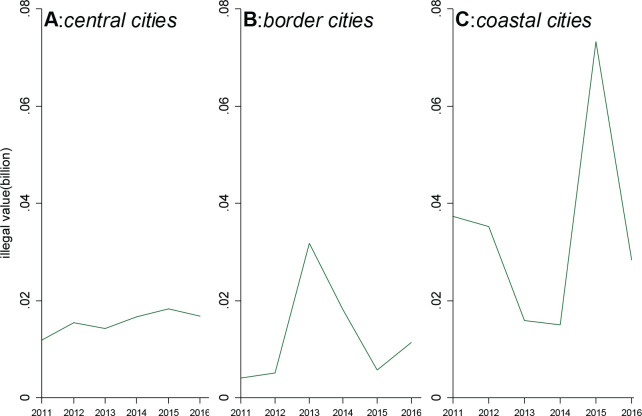
Aggregate annual SIVST over time for prefectures grouped by location

Geography might play an important role in cigarette smuggling. Figure 2 shows various change patterns of SIVST over time among different types of cities. We classified all prefectures into three groups – central cities, border cities, and coastal cities. Border cities encompass prefectures with edges adjacent to another nation; coastal cities consist of prefectures situated at the interface between land and sea, while the remaining prefectures were categorized as central cities. The diverse change patterns in economic crime are apparent across different groups of cities. Figure 2A shows a gradual and slow increase in SIVST from 2011 to 2016 with minimal variance.

Conversely, Figure 2B reveals a distinct pattern in border cities compared to central cities, with SIVST experiencing a sudden surge in 2013. Figure 2C presents the SIVST trend in coastal cities, notably showing a substantial spike in 2015. The national SIVST of coastal cities in 2015 was nearly four times that of 2014. Overall, the volume of smuggling in coastal cities significantly surpasses that in other areas, which means that coastal cities might be the most impacted by the 2015 cigarette excise tax hikes.

### Analytical approach

Many factors influence economic crime, such as economic conditions, cultural differences, and family ties^6,17,18^. It is imperative to deal with the endogeneity problem posed by omitted variables. Generally, the DID approach is one of the ways to address potential endogeneity concerns. Still, a major challenge in our analysis is that the counterfactual outcomes in the absence of the policy are unobserved. To solve this problem, we follow Mian and Sufi^19^ to form counterfactual outcomes based on cross-sectional variation across prefectures in their exposure to the 2015 tobacco tax policy. The measure of exposure depends on SIVST in 2014 (one year before the policy shock) because it reasonably reflects different tax incentives across cities. In other words, after the policy shock, criminals have much more incentive to smuggle tobacco in a prefecture with a higher SIVST in 2014. Referring to the studies of Beck et al.^20^ and Gehrsitz et al.^21^, the baseline model can be written as:

$$SIVST_{\text{it}}=a+\beta \left(exposure\times dummy_{2015}\right)+X^{'}\gamma +\eta_{t}+u_{i}+\varepsilon_{\text{it}}$$ 1

where *i* is the index for prefecture, and *t* denotes year. The variable *dummy*_2015_ is a dummy variable, equal to 1 if the observation is in the period after the tobacco tax shock (i.e. in the period 2015–2016) and 0 otherwise. SIVST_it_ is the variable explained in this model, reflecting the economic crime situation of prefecture *i* in year *t*. Because large prefectures naturally have more population and demand for tobacco, to construct a reasonable variable (*exposure*_i_) that could validly reflect the degree of exposure to the 2015 tobacco tax policy, we standardized SIVST by the population in each prefecture *i* in 2014. Specifically, *exposure*_i_ is constructed as below:

$$exposure_{\text{i}}=SIVST_{\text{i2014}}/population_{\text{i2014}}$$ 2

Additionally, we include prefecture-level controls, *X* represents a set of control variables, including the Log wage of all workers, Log per capita GDP, etc. Parameters *u*_i_ *and η*_t_ control for city-fixed and year-fixed effects, respectively, and *ε*_it_ is the residual term. Overall, there are 1646 prefecture-level observations in our analysis sample.

## RESULTS

Table 1 presents summary statistics (means and standard deviations) for the variables used in Equation 1. SIVST is the seized-illegal value of smuggling tobacco of prefectures. Log SIVST is the natural logarithm value of SIVST. SIVST/GDPis SIVST of a prefecture normalized by the GDP of the prefecture. Referring to Zwick and Mahon^22^, the rest of the control variables, such as wage, per capita GDP, education expenditure, and population, are also linearized via a logarithmic transformation (log value). The table shows the summary statistics across three periods: Full sample, 2011–2014, and 2015–2016. The mean values of all variables increased from 2011 to 2016, but there is a noticeable difference between the explained variable and the other variables. SIVST, Log SIVST, and SIVST/GDP experience relatively large increases. Variables such as the log wage of all workers, log per capita GDP and log education expenditure show a minor increase in mean values over time. This could suggest a general increase in economic development. The log population remains relatively stable, indicating little change in population size over the years covered by the data.

**Table 1:** Summary statistics of cigarette crime and other economic variables, 2011–2016, China (N=1680)

*Variables*	*Full sample*		*2011–2014*		*2015–2016*	
	*Mean*	*SE*	*Mean*	*SE*	*Mean*	*SE*
SIVST	13.02	19.92	12.15	18.59	14.71	22.27
Log SIVST	1.82	1.29	1.76	1.28	1.93	1.30
SIVST/GDP	1.23	5.76	1.18	5.43	1.32	6.36
Log wage of all workers	14.20	0.88	14.08	0.86	14.48	0.87
Log per capita GDP	10.59	0.56	10.52	0.58	10.71	0.51
Log education expenditure	12.95	0.68	12.86	0.66	13.13	0.68
Log population	5.85	0.69	5.85	0.68	5.85	0.69

Units of SIVST, the wage of all workers, SIVST/GDP, per capita GDP, education expenditure, and population are 1 million Yuan, 10 thousand Yuan, Yuan/Yuan, 1 Yuan per person, 10 thousand Yuan, and 10 thousand people, respectively. RMB: 1000 Chinese Yuan Renminbi about US$140, current exchange. SIVST is winsorized at the 1st and 99th percentiles of their distributions. SE: standard error. Summary statistics of control variables in the natural scale are reported in the Supplementary file.

### Baseline results

Table 2 reports the OLS estimate without introducing control variables and year fixed effect. Column (1) shows the relationship between tax exposure and economic crime. The estimated coefficient of interaction between *exposure* and *dummy*_2015_ is 0.32, which is economically large and significantly different from 0. After adding year fixed effect into the regression, the impact of tax still exists. Regression results are presented in column (3) when both the fixed effects and control variables are included. For every 1-unit increase in per capita illicit trade in cigarettes in the region in 2014, the local illicit trade in cigarettes increased by 0.25 units after 2015. These coefficients of interaction terms are similar. This outcome suggests that raising the tax rate on tobacco obviously increases criminal activities.

**Table 2:** Effect of 2015 GST increase on economic crime in China (N=1642)

	*(1)*	*(2)*	*(3)*
	*SIVST*	*SIVST*	*SIVST*
*Exposure*×*dummy*_2015_	0.318***	0.262***	0.253***
	(4.90)	(4.35)	(4.16)
Log wage of all workers			2.043
			(0.88)
Log per capita GDP			1.328
			(0.43)
Log education expenditure			5.177*
			(1.90)
Log population			2.776
			(0.37)
Constant	12.61***	12.52***	-111.2*
	(148.04)	(16.50)	(-1.82)
CITY_FE	Yes	Yes	Yes
YEAR_FE	No	Yes	Yes
R^2^	0.02	0.03	0.03
N	1646	1646	1642

The t statistics are in parentheses. Standard error clustered at the prefecture level. *, **, *** statistically distinct from 0 at the 10%, 5%, 1% significance level, respectively. CITY_FE and YEAR_FE indicate city-fixed effects and year-fixed effects, respectively. R^2^ is a statistic that reflects the model’s goodness of fit as the ratio of the regression sum of squares to the total sum of squares. N denotes the sample size for the regression analysis.

### Robustness and heterogeneity

To ensure that the above outcome is valid, we performed a variety of robustness checks. In Table 3, we look at the sensitivity of our estimates when using different specifications. First, we changed the explained variable to a log specification [column (1)]. It showed that the coefficient of the interaction term is 0.0098 – the tax hike’s impact on economic crime is statistically significant. Secondly, the amount of local cigarette economic offenses as a share of local GDP similarly captures the severity of local cigarette offenses, so we used SIVST/ GDP as a new dependent variable to replace SIVST in the regression. The coefficients on the interaction terms in Table 3 were all significantly positive, consistent with the results of the benchmark regression.

**Table 3:** Robust test: effect of 2015 GST increase on economic crime in China (N=1642)

	*(1)*	*(2)*
	*Log SIVST*	*SIVST/GDP*
*Exposure*×*dummy*_2015_	0.00982**	0.000323***
	(2.35)	(3.84)
Log wage of all workers	0.305**	-0.0000978
	(2.36)	(-0.05)
Log per capita GDP	0.0596	0.00235
	(0.28)	(1.04)
Log education expenditure	0.312	0.00164
	(1.55)	(0.66)
Log population	0.997	-0.00101
	(1.50)	(-0.18)
Constant	-12.89***	-0.0286
	(-2.64)	(-0.61)
CITY_FE	Yes	Yes
YEAR_FE	Yes	Yes
R^2^	0.05	0.04
N	1642	1642

The t statistics are in parentheses. Standard error clustered at the prefecture level. *, **, *** statistically distinct from 0 at the 10%, 5%, 1% significance level, respectively. CITY_FE and YEAR_FE indicate city-fixed effects and year-fixed effects, respectively. R^2^ is a statistic that reflects the model’s goodness of fit as the ratio of the regression sum of squares to the total sum of squares. N denotes the sample size for the regression analysis.

After implementing a policy, if the policy effects exhibit a lag, it usually leads to underestimating the results rather than overestimating. To determine whether our study is affected by a lag problem in policy effects, we examined tobacco smuggling data for the first and second years following the tax rate increase 2015. At first, we tested the first-year effect of GST increase by deleting 2016 observations from our sample and deleting 2015 observations from our sample to test the second-year effect. Results are shown in columns (1) and (2) of Table 4. Our analysis reveals that the policy’s impact is most significant in the first year of implementation [see column (1)], obviously higher than the average, but it diminishes in the subsequent years [see column (2)]. This indicates that the policy’s effects are not long-lasting, with a noticeable reduction over time.

**Table 4:** The effect of GST increase on SIVST among cities after deleting observations of each year in China, respectively

	*(1)*	*(2)*	*(3)*	*(4)*	*(5)*	*(6)*
*Deleted year*	*2016*	*2015*	*2014*	*2013*	*2012*	*2011*
*Exposure*×*dummy*_2015_	0.412***	0.147**	0.296***	0.224***	0.197***	0.276***
	(3.40)	(2.29)	(4.95)	(3.82)	(3.21)	(2.92)
Log wage of all workers	0.253	1.938	3.560	2.361	1.250	1.654
	(0.08)	(0.84)	(1.27)	(1.01)	(0.51)	(0.59)
Log per capita GDP	2.354	1.915	-1.775	9.726**	1.130	-0.686
	(0.66)	(0.59)	(-0.50)	(2.39)	(0.37)	(-0.23)
Log education expenditure	3.999	3.913	6.566**	2.801	6.013**	6.225**
	(1.25)	(1.43)	(2.20)	(0.85)	(2.14)	(2.38)
Log population	-1.045	1.248	3.193	2.439	4.472	4.300
	(-0.06)	(0.16)	(0.39)	(0.30)	(0.59)	(0.59)
Constant	-59.84	-90.80	-120.0*	-170.8**	-118.7**	-109.6*
	(-0.54)	(-1.44)	(-1.86)	(-2.53)	(-2.00)	(-1.79)
CITY_FE	Yes	Yes	Yes	Yes	Yes	Yes
YEAR_FE	Yes	Yes	Yes	Yes	Yes	Yes
R^2^	0.03	0.02	0.04	0.03	0.03	0.04
N	1368	1370	1368	1367	1369	1368

The t statistics are in parentheses. Standard error clustered at the prefecture level. *, **, *** statistically distinct from 0 at the 10%, 5%, 1% significance level, respectively. CITY_FE and YEAR_FE indicate city-fixed effects and year-fixed effects, respectively. R^2^ is a statistic that reflects the model’s goodness of fit as the ratio of the regression sum of squares to the total sum of squares. N denotes the sample size for the regression analysis.

Second, during the sample period, no specific policies or major events directly targeting tobacco smuggling existed. However, the government implemented two significant tax incentives, which may have influenced the motivation for economic crimes by altering supply and demand dynamics in the commodity market. Putting the Program of Replacing Business Tax with Value-added Tax (enacted in 2012) and Accelerated Depreciation Policy of Fixed Assets (enacted in 2014) into consideration, we introduce the interaction variables (*BusinessTax*×*dummy*_2012_, *DepreciationPolicy*×*dummy*_2014_) into regression models. *BusinessTax* is defined as the percentage of the business tax in total tax revenue of cities. *DepreciationPolicy* is defined as the fixed assets of firms affected by the policy in terms of the total fixed assets of firms. The two variables reflect the degree to which these policies affect cities. The variables *dummy*_2012_ and *dummy*_2014_ are dummy variables, which are defined similarly to *dummy*_2015_. We re-estimate these models, and Table 5 reports the results. The significant level of all coefficients of the key interaction term remains unchanged. Further robustness checks, such as testing pre-event trends, introducing other control variables, employing the difference-in-difference method, conducting placebo tests, re-defining key explanatory variables, and so on, also validate our conclusions (details can be found in the Supplementary file).

**Table 5:** The effect of GST increase on SIVST after controlling the influence of other policies (N=1642)

	*(1)*	*(2)*	*(3)*
*Exposure*×*dummy*_2015_	0.252***	0.253***	0.252***
	(4.16)	(4.19)	(4.17)
*BusinessTax*×*dummy*_2012_	-5.250		-5.250
	(-0.54)		(-0.56)
*Depreciation Policy*×*dummy*_2014_		-2.576	-2.443
		(-1.12)	(-1.05)
Log wage of all workers	2.267	2.043	2.267
	(0.97)	(0.89)	(0.97)
Log per capita GDP	1.123	1.328	1.123
	(0.37)	(0.40)	(0.33)
Log education expenditure	5.340*	5.177*	5.340*
	(1.95)	(1.66)	(1.71)
Log population	3.011	2.776	3.011
	(0.40)	(0.25)	(0.27)
Constant	-115.6*	-111.2	-115.6
	(-1.85)	(-1.50)	(-1.55)
CITY_FE	Yes	Yes	Yes
YEAR_FE	Yes	Yes	Yes
R^2^	0.03	0.03	0.03
N	1642	1642	1642

The t statistics are in parentheses. Standard error clustered at the prefecture level. *, **, *** statistically distinct from 0 at the 10%, 5%, 1% significance level, respectively. CITY_FE and YEAR_FE indicate city-fixed effects and year-fixed effects, respectively. R^2^ is a statistic that reflects the model’s goodness of fit as the ratio of the regression sum of squares to the total sum of squares. N denotes the sample size for the regression analysis.

As shown in Figure 2, the change pattern of SIVST over time differs among central, border, and coastal cities. We observe that only SIVST in coastal prefectures jumped in 2015, and there is no such jump in central and border prefectures. Hence, the observed national rise of SIVST in 2015 in Figure 1 is primarily driven by the SIVST rise of coastal cities. To provide more compelling evidence on the heterogeneity effect of tax hikes, we create the *coastal* variable as a dummy variable, which equals one if prefectures belong to coastal cities and 0 if prefectures are central cities. Subsequently, we interact *coastal* with *exposure*, *dummy*_2015_, and incorporate this new interaction term into the regression model (Equation 1). These regression results are shown in Supplementary file Table A4. For all three columns, the triple interaction is significantly positive. Therefore, the impact of taxes on economic crime is obviously stronger in coastal cities. Further, we create a variable *border* as a dummy variable, which equals one if a prefecture belongs to a border city and 0 if a prefecture is a central city. Supplementary file Table A5 presents the regression results with the triple interaction (*exposure×dummy*_2015_×*border*). Columns (1) to (3) show that the tax hike’s impact on economic crime is roughly similar between border and central cities.

## DISCUSSION

This is the first study focusing on the impact of the GST on cigarettes (cigarette excise tax) on cigarette economic crime in China. Measuring the scale of cigarette crime has always been a challenge. Most previous literature has measured the illicit trade in cigarettes in an indirect way^23-25^. For example, Nguyen et al.^24^ used a gap method to estimate the difference between domestically tax-paid cigarette sales and gross domestic consumption in Vietnam. In this article, the crime of cigarettes refers to a large-scale lucrative activity involving illegal transportation, distribution, and sale of large consignments of cigarettes, generally avoiding all or partial taxes. Compared to previous studies, we use new data and research methods. Specifically, this work uses the value of illicit trade in cigarettes seized by the government to measure the scale of cigarette economic crime. In addition, for the first time, we use Chinese prefecture-level panel data from 2011–2016 and a DID approach to analyze the relationship between cigarette excise taxes and cigarette smuggling. This complements the existing theoretical and narrative studies on the link between tax and economic crime. In addition, our findings are different from those of previous studies. Delipall^26^ argued that higher cigarette taxes would affect cigarette smuggling differently under different tax structures, with higher *ad valorem* taxes exacerbating cigarette smuggling^26^. The Tax Foundation of United States finds a strong positive relationship between cigarette smuggling and tax rates across the states^27^. In contrast, our empirical results show that a nationwide tax increase policy does not lead to an increase in cigarette smuggling in all parts of the cities, but it does lead to an increase in cigarette smuggling in coastal cities.

The primary mechanism at play involves the alteration of incentives and opportunities within the market. When the government raises the sales tax on cigarettes, the retail price of these products increases correspondingly. The Chinese 2015 policy at least doubled the cigarette consumption tax, obviously raising the market price of cigarettes. This price hike can lead to higher profit margins for offenders engaged in the illicit trade of cigarettes, who can offer these products at lower prices than legal vendors by evading taxes. Consequently, the potential financial gains from smuggling become more attractive, incentivizing economic crimes related to tobacco. Moreover, higher cigarette prices might reduce legal demand, but it can simultaneously create an underground market where consumers seek cheaper alternatives. This demand shift can bolster the operations of organized crime groups involved in smuggling and selling untaxed cigarettes, further embedding economic crime in the supply chain.

This study employs a difference-in-difference (DID) method to analyze the impact of the increase in the goods and services tax (GST) on economic crime, specifically focusing on the illicit tobacco trade in China following a tobacco excise tax hike in 2015. The study uses panel data from 2011–2016, combining prefecture-level socio-economic data with smuggling data.

### Limitations

While our results are novel, there are limitations to the current analysis, as data are only available up to 2016, and the State Tobacco Monopoly Administration stopped disclosing relevant data after that year. Moreover, although our analysis controls for other policies, other unaccounted factors may influence the results.

## CONCLUSIONS

This study sheds light on the previously overlooked relationship between GST increase and economic crime, specifically focusing on the impact of cigarette excise tax in China. Economic crime, encompassing various illicit activities, has become a growing concern globally due to its detrimental effects on economic development, financial stability, and social cohesion. While previous research has extensively explored the influencing factors of economic crime, the role of GST in shaping criminal behavior has been largely ignored. Our findings suggest that while raising the excise tax on cigarettes promotes cigarette smuggling, it primarily promotes cigarette smuggling in coastal cities, not all cities. Based on the results of the above empirical analysis, we believe that considering that cigarette smuggling weakens the effect of raising taxes and controlling cigarettes, governments need to strengthen the regulation and penalties of cigarette smuggling while raising cigarette taxes.

## Supplementary Material



## Data Availability

The data supporting this research are available from the authors on reasonable request.
