# Supplementary material for: The impact of goods and services tax increase on economic crime: Evidence from China’s tobacco tax hike
Source: Tob Induc Dis. 2025 Jan 17;23:10.18332/tid/197330. doi: 10.18332/tid/197330 (PMC11740871; doi:10.18332/tid/197330)
Supplement: Supplementary file 1 [file TID-23-03-s1.pdf]

Supplementary material

Supplementary file Table A0. Summary statistics of control variables in natural scale (N=1680)

	Obs	Mean	Std.	dev.	Min
Panel A: Full sample					
wage of all workers	1,677	2284069	2872163	174391	2.60e+07
per capita GDP	1,680	46675.61	30192.88	6457	290477
education expenditure	1,680	525758.5	378507.2	20048	3219820
population	1,680	424.5666	253.5467	19.5	1399
Panel B: 2011-2014					
wage of all workers	1,115	1996167	2474037	174391	2.30e+07
per capita GDP	1,118	44350.57	30507.6	6457	290477
education expenditure	1,118	474200.5	326802.7	20048	2539473
population	1,118	423.2512	251.2843	19.5	1238.5
Panel C: 2015-2016					
wage of all workers	562	2855264	3463958	213157	2.60e+07
per capita GDP	562	51300.87	29034.36	10987	215488
education expenditure	562	628324.2	447546.2	35925	3219820
population	562	427.1834	258.1945	20.25	1399

Notes: The units of the variables are as follows: Total wages: 10,000 yuan; GDP per capita: yuan; Education expenditure: 10,000 yuan; Total population: 10,000 people.

The observed policy effect of GST increase might not be attributable solely to the policy itself but might be also influenced by other observed and unobserved factors. To addressed this potential threat we additionally employ several different methods, and the results demonstrate that our conclusions are still robust.

First, when employing the DID method, the outcome variable (SIVST) among cities should be indifference before the implementation of the policy. The parallel trend test graph shows that before the policy implementation, cities with large per capita smuggling scales does not exhibit significant larger smuggling scales compared to cities with smaller per capita smuggling scales. This means that after the policy implementation, the significant difference of smuggling between cities stems from the GST increase.

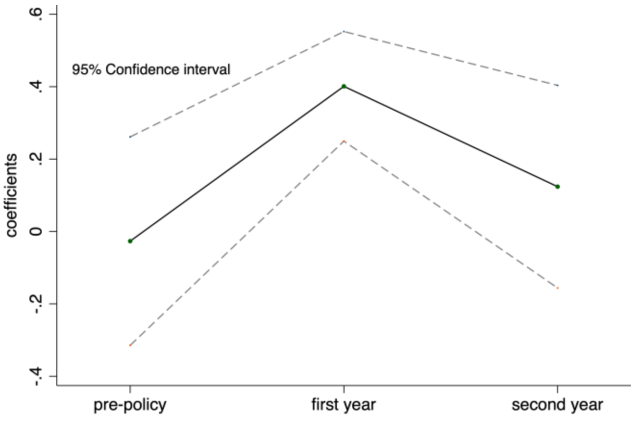

Supplementary file Figure A1. The difference of SIVST among cites before and after the policy implementation

Second, in a linear panel event-study design, unobserved confounds may be related both to the outcome and to the policy variable of interest. To address this problem, a placebo test and a Difference-in-Difference-in-Difference (DDD) method are used. By using fake different cut-off points of 2013 for the policy implementation period, we redefine the key explanatory variable as $Exposure*dummy_{fake}$ and re-estimate model. Results are shown in column (1) of Table A1, and the coefficient of the interaction term is no longer significant. This finding ensures that the observed effects are not due to other policy or confounding factors. By classifying sample into two groups based on cigarette sales of cities in 2014, we define a dummy variable *Sale* that equals 1 if cities with higher cigarette sales, otherwise, 0. We introduce the triple interaction of *Sale* and original key explanatory variable into regression model to eliminate the possible pre-event trend caused by unobserved factors. Result in column (2) shows that coefficient of the triple interaction is significantly at 1%, which further validates our conclusions.

Supplementary file Table A1. The effect of GST increase on SIVST among cities after delete observations of each years, respectively

SIVST	(1)	(2)
*Exposure*dummy*$_{fake}$	43.66	
	(1.09)	
*Exposure*dummy*$_{2015}$**Sale*		0.277***
		(3.02)
Log wage of all workers	2.123	2.092
	(0.79)	(0.90)
Log per capita GDP	1.905	1.664
	(0.58)	(0.53)
Log education expenditure	5.050*	4.759*
	(1.73)	(1.71)
Log population	2.189	3.062
	(0.29)	(0.42)
constant	-113.3*	-111.8*
	(-1.80)	(-1.83)
CITY_FE	YES	YES
YEAR_FE	YES	YES
R^2	0.03	0.03
N	1622	1642

Notes: the t statistics are in parentheses. Standard error clustered at the prefecture level. *, **, *** statistically distinct from 0 at the 10%, 5%, 1% significance level, respectively. CITY_FE and YEAR_FE indicate city-fixed effects and year-fixed effects, respectively. R2 is a statistic that reflects the model's goodness of fit as the ratio of the regression sum of squares to the total sum of squares. N denotes the sample size for the regression analysis.

Third, employment situation matters to economic crimes, thus it is necessary to consider them as control variables. Specifically, we incorporate two variables *Employment* (ratio of employment

to total population) and *Unemployment* (ratio of unemployment to total population) reflecting the employment situation of cities as control variables in the model. Results are reported in Table A2. Columns (1) to (3) show positively significant coefficients of the interaction term. That is, our conclusions are unchanged under the controlling of employment situation.

Supplementary file Table A2. The effect of GST increase on SIVST after controlling employment situation and improving the definition of key explanatory variable

SIVST	(1)	(2)	(3)	(4)	(5)
*Exposure*dummy*$_{2015}$	0.252***	0.267***	0.266***		
	(4.15)	(4.50)	(4.47)		
*Exposure*dummy*$_{new}$				39.48*	59.18***
				(1.85)	(2.74)
Log wage of all workers	3.753	2.260	3.993	1.698	4.668
	(1.33)	(0.96)	(1.39)	(0.70)	(1.60)
Log per capita GDP	1.631	0.819	1.169	3.347	4.105
	(0.52)	(0.26)	(0.38)	(1.04)	(1.29)
Log education expenditure	5.068*	5.766**	5.557*	4.919*	5.083*
	(1.88)	(1.97)	(1.92)	(1.75)	(1.69)
Log population	1.342	1.766	0.512	1.493	-3.588
	(0.18)	(0.23)	(0.07)	(0.20)	(-0.49)
Employment	-14.04**		-14.24**		-24.26***
	(-2.17)		(-2.15)		(-3.51)
Unemployment		0.00308	0.00354		0.00238
		(0.41)	(0.48)		(0.31)
constant	-126.6**	-110.5*	-126.6**	-116.6*	-135.3**
	(-2.06)	(-1.79)	(-2.04)	(-1.85)	(-2.12)
CITY_FE	YES	YES	YES	YES	YES
YEAR_FE	YES	YES	YES	YES	YES
R^2	0.03	0.04	0.04	0.03	0.03

N	1642	1622	1622	1642	1622

Notes: the t statistics are in parentheses. Standard error clustered at the prefecture level. *, **, *** statistically distinct from 0 at the 10%, 5%, 1% significance level, respectively. CITY_FE and YEAR_FE indicate city-fixed effects and year-fixed effects, respectively. R2 is a statistic that reflects the model's goodness of fit as the ratio of the regression sum of squares to the total sum of squares. N denotes the sample size for the regression analysis.

Finally, considering the correlation between pre-policy SIVST and post-policy SIVST, and to demonstrate that our findings are insensitive to the construction way of key explanatory variable, we use a more robust indicator to substitute the original key explanatory variable. We improve the original key explanatory variable by substituting the cigarette sales amount of cities in 2014 for $SIVST_{2014}$ in equation (2) of manuscript for two reasons: 1) The amount of cigarette sales in cities, to some extent, is exogenous with SIVST of cities. The insignificant coefficients of the interaction term and SIVST in Table A3 show that not only SIVST is uncorrelated with cigarette sales, but also the 2015 cigarette tax policy affect little on cigarette sales. This indicates that cigarette sales are independent of smuggling activities and the increase in GST, thereby satisfying the exogeneity requirements for the variable construction of policy shock. 2) Sales indicate market demand and reflect both the scale and difficulty of profiting from smuggling. Following the tax increase, cities with higher sales offer offenders greater profit opportunities through smuggling. Therefore, sales volume directly reflects the intensity of the impact of policy. Thus, it is reasonable to use the cigarette sales of cities as an proxy variable of policy. Coefficients of the new key explanatory variable are shown in columns (4) and (5) of Table A2. The results demonstrate that our conclusions are still robust.

Supplementary file Table A3. The relationship between SIVST and cigarette sales

Sales	(1)	(2)
$Exposure*dummy_{2015}$	0.0182	
	(0.74)	
SIVST		0.00262
		(0.15)

Log wage of all workers	5.231***	5.168***
	(3.36)	(3.36)
Log per capita GDP	4.166**	3.720*
	(2.07)	(1.78)
Log education expenditure	4.142***	4.330***
	(3.00)	(3.07)
Log population	9.754	6.881
	(1.09)	(0.83)
constant	-197.9***	-178.1***
	(-3.75)	(-3.70)
CITY_FE	YES	YES
YEAR_FE	YES	YES
R^2	0.41	0.42
N	1673	1642

Notes: the *t* statistics are in parentheses. Standard error clustered at the prefecture level. *, **, *** statistically distinct from 0 at the 10%, 5%, 1% significance level, respectively. CITY_FE and YEAR_FE indicate city-fixed effects and year-fixed effects, respectively. R^2 is a statistic that reflects the model's goodness of fit as the ratio of the regression sum of squares to the total sum of squares. N denotes the sample size for the regression analysis.

Supplementary file Table A4. Heterogeneity effect of GST increase on economic crime between central and coastal cities (N=1545)

	(1)	(2)	(3)
	SIVST	*SIVST*	*SIVST*
*Exposure*dummy$_{2015}$*coastal*	1.511**	1.348*	1.305*
	(2.01)	(1.77)	(1.68)
*Exposure*dummy$_{2015}$*	0.287***	0.253***	0.246***
	(3.86)	(3.32)	(3.19)
Log wage of all workers			1.492

			(0.60)
Log per capita GDP			2.288
			(0.73)
Log education expenditure			4.174
			(1.43)
Log population			-0.802
			(-0.11)
constant	12.80***	13.02***	-79.60
	(76.74)	(16.12)	(-1.28)
CITY_FE	YES	YES	YES
YEAR_FE	NO	YES	YES
R^2	0.03	0.04	0.04
N	1549	1549	1545

Notes: the *t* statistics are in parentheses. Standard error clustered at the prefecture level. *, **, *** statistically distinct from 0 at the 10%, 5%, 1% significance level, respectively. CITY_FE and YEAR_FE indicate city-fixed effects and year-fixed effects, respectively. R^2 is a statistic that reflects the model's goodness of fit as the ratio of the regression sum of squares to the total sum of squares. N denotes the sample size for the regression analysis.

Supplementary file Table A5. Heterogeneity effect of GST increase on economic crime between central and border cities (N=1371)

	(1)	(2)	(3)
	SIVST	*SIVST*	*SIVST*
*Exposure*dummy*$_{2015}$**border*	-0.0489	-0.0366	-0.0338
	(-0.60)	(-0.45)	(-0.39)
*Exposure*dummy*$_{2015}$	0.287***	0.256***	0.244***
	(3.86)	(3.46)	(3.22)
Log wage of all workers			3.433
			(1.31)

Log per capita GDP			3.400
			(1.04)
Log education expenditure			4.696**
			(2.04)
Log population			1.114
			(0.20)
constant	11.30***	10.66***	-137.1***
	(130.33)	(14.38)	(-2.80)
CITY_FE	YES	YES	YES
YEAR_FE	NO	YES	YES
R^2	0.02	0.03	0.03
N	1375	1375	1371

Notes: the *t* statistics are in parentheses. Standard error clustered at the prefecture level. *, **, *** statistically distinct from 0 at the 10%, 5%, 1% significance level, respectively. CITY_FE and YEAR_FE indicate city-fixed effects and year-fixed effects, respectively. R^2 is a statistic that reflects the model's goodness of fit as the ratio of the regression sum of squares to the total sum of squares. N denotes the sample size for the regression analysis.

© 2025 Zhang X. and Zhang Z.
